# Cross national comparison of memory function before and after a cancer diagnosis in the United States and England

**DOI:** 10.1007/s11764-026-01969-8

**Published:** 2026-01-17

**Authors:** Ashly C. Westrick, Lauren P. Wallner, Alden L. Gross, Kenneth M. Langa, Lindsay C. Kobayashi

**Affiliations:** 1 Department of Epidemiology and Biostatistics, Michigan State University College of Human Medicine, East Lansing, MI USA; 2 Department of Public Health Sciences, Henry Ford Health, Detroit, MI USA; 3 Department of Internal Medicine, University of Michigan Medical School, Ann Arbor, MI USA; 4 Department of Epidemiology, School of Public Health, University of Michigan, Ann Arbor, MI USA; 5 Department of Epidemiology, Johns Hopkins Bloomberg School of Public Health, Baltimore, MD USA; 6 Survey Research Center, Institute of Social Research, University of Michigan, Ann Arbor, MI USA; 7 Rogel Cancer Center, University of Michigan, Ann Arbor, MI USA; 8 Institute for Healthcare Policy and Innovation, University of Michigan, Ann Arbor, USA

**Keywords:** Aging, Cross-national comparison, Cancer survivors, Cognitive decline

## Abstract

**Objectives:**

The long-term memory aging of middle-aged and older cancer survivors is not fully understood and is largely limited to the United States (US) context. We compared long-term memory trajectories before and after an incident cancer diagnosis with the memory trajectories of similarly aged cancer-free adults in the US and England.

**Methods:**

Incident cancer diagnosis and memory (immediate and delayed recall) were assessed during biennial interviews in the US Health and Retirement Study (HRS; n = 13,037) and the English Longitudinal Study of Ageing (ELSA; n = 8,579) from 2002 to 2018. Within country, multivariable adjusted linear mixed-effects models estimated the standardized memory trajectories in participants with and without cancer.

**Results:**

Prior to diagnosis, cancer survivors had a memory advantage compared to cancer-free individuals, within each country (difference for cancer survivors in the US: 0.10 SD units [95% CI: 0.05, 0.14] and in England: 0.14 SD units [95% CI: 0.07, 0.20]). Cancer survivors in the US experienced an acute decline in memory function immediately following diagnosis (-0.08 SD units; 95% CI: -0.11, -0.04), which was not experienced by cancer survivors in England (0.01 SD units; 95% CI: -0.04, 0.07). Long-term post-diagnosis memory trajectories were similar for cancer survivors and cancer-free participants alike in both countries.

**Conclusion:**

The acute decline in memory function associated with a new cancer diagnosis among cancer survivors in the US, but not England, warrants future studies to investigate explanations for this cross-national difference, such as differences in healthcare systems and treatment.

**Implications for Cancer Survivors:**

Results can generate hypotheses to identify contextual risk factors to improve cognitive aging of cancer survivors.

**Supplementary Information:**

The online version contains supplementary material available at https://doi.org/10.1007/s11764-026-01969-8.

## Introduction

The United States (US) and England are both experiencing rapidly aging populations [1]. With the improvements in cancer screening, diagnoses, and treatment, it is estimated that by 2030 the number of cancer survivors will double in both countries [2, 3]. Cancer survivors experience a myriad of long-term effects from cancer and its treatments, including memory changes [4–6]. Previous clinical-based research has found short-term cognitive declines after cancer treatment [4, 7] while larger population-based studies consistently report an inverse association between incident cancer and subsequent dementia risk [8, 9]. However, as most previous research on cancer-related cognitive impairment has been largely limited to the US, it is unknown if cognitive aging trajectories differ in other countries. This gap limits our understanding of cognitive aging, as data from other countries provide variation in key contextual factors that could be targeted to improve population health and reduce health inequalities [10].

The US and England are two Western, high-income countries that share many similarities including language and economic systems [11]. However, they differ in terms of healthcare systems and social safety net programs, which could confer different aging trajectories of older cancer survivors in both countries. Previous cross-national comparisons of the health of older adults in the US and England have found that older adults in the US have a higher prevalence of comorbidities, including hypertension, diabetes, and heart disease than their counterparts in England [12]. Despite the higher prevalence of cardiovascular risk factors and disease which are associated with cognitive impairment and decline, Langa et al., reported that US adults had a cognitive advantage compared to adults in England [13]. However, to the best of our knowledge, no study has compared the long-term memory aging trajectories of older cancer survivors in the US and England. The primary goals of this study were to 1) compare the long-term memory aging trajectories of older cancer survivors prior to, at the time of, and after a cancer diagnosis, relative to the long-term memory aging trajectories of age-matched cancer-free individuals, both in the US and England, and 2) to determine if these trajectories differ by country. We hypothesized that, due to differences in social safety nets between these two countries, there is an acute decline in memory function associated with a new cancer diagnosis among cancer survivors in the US but not England.

## Methods

### Study sample

*The US Health and Retirement Study (HRS).*The US HRS is a nationally representative longitudinal study of > 30,000 adults aged > 50 years in the United States, with biennial study interviews beginning in 1992 [14].

*The English Longitudinal Study of Ageing (ELSA).*The ELSA is a nationally representative longitudinal study of > 11,000 adults aged > 50 years in England, with biennial study interviews beginning in 2002 [15]. Its design and measures are harmonized with the HRS.

**Fig. 1:**
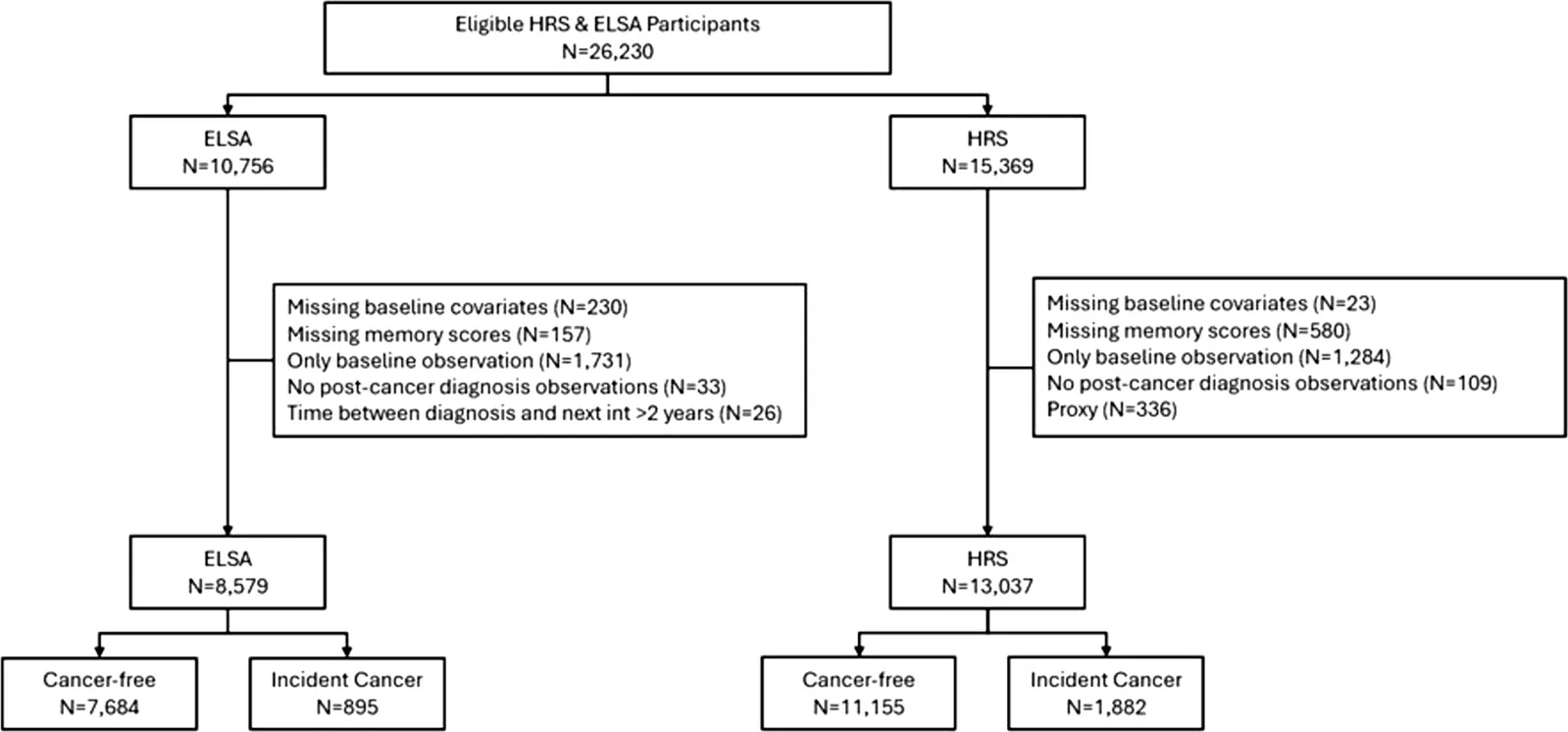
Sample flow chart for the US health and retirement study and the english longitudinal study on ageing

In both cohorts, we used the 2002 study interview as the baseline and included follow-up data through 2018. We restricted the study sample to participants with no history of cancer who were 50 years or older at baseline, and who had complete baseline covariate data. After excluding non-eligible participants (Fig. 1), the final analytic sample was 21,616 individuals (13,037 from HRS; 8,579 from ELSA) with 2,777 (12.5%) individuals with an incident cancer diagnosis over the follow-up (1,882 from HRS; 895 from ELSA). Supplemental Table 1 compares the characteristics of those individuals included and excluded from our analysis.

**Table 1:** Baseline Characteristics of the Sample, overall and by country, in the 2002 wave of the US Health and Retirement Study (HRS; n = 13,037) & the English Longitudinal Study on Ageing (ELSA; n = 8579)

	Total N = 21,616	ELSA N = 8579	HRS N = 13,037
Age, *years, mean (SD)*	66.0 (9.50)	64.0 (9.72)	67.3 (9.12)
Memory, *mean (SD)*	9.93 (3.54)	9.68 (3.46)	10.1 (3.59)
Age- and comorbidity- adjusted memory, *mean (95% CI)*	9.85 (9.60, 9.93)	10.2 (10.1, 10.2)	9.10 (9.04, 9.16)
Sex/Gender, *n (%)*			
Male	9110 (42.1)	3896 (45.4)	5214 (39.9)
Female	12,506 (57.9)	4683 (54.6)	7823 (60.1)
Race, *n (%)*			
White	19,117 (88.4)	8381 (97.7)	10,736 (82.4)
Non-White	2499 (11.6)	198 (2.32)	2301 (17.7)
Educational Attainment, *n (%)*			
Less than high school	7580 (35.1)	4510 (52.6)	3070 (23.6)
High school	6328 (29.3)	1476 (17.2)	4852 (37.2)
Some college	4203 (19.4)	1556 (18.1)	2647 (20.3)
College and above	3505 (16.2)	1037 (12.1)	2468 (18.9)
Marital Status, *n (%)*			
Married/Partnered	14,830 (68.6)	6049 (70.5)	8781 (67.4)
Divorced/Separated	2143 (9.91)	781 (9.10)	1362 (10.5)
Widowed	3907 (18.1)	1345 (15.7)	2562 (19.7)
Never Married	736 (3.40)	404 (4.7)	332 (2.55)
Comorbidities, *yes, n (%)*			
Hypertension	8759 (40.5)	2405 (28.0)	6354 (48.7)
Diabetes	2574 (11.9)	548 (6.39)	2026 (15.5)
Stroke	1103 (5.10)	286 (3.33)	817 (6.27)
Heart disease	3832 (17.7)	1284 (15.0)	2548 (19.5)
Self-report any cancer treatment, *yes, n (%)*	1807 (65.1)	779 (89.5)	1028 (54.6)

SD, standard deviation. Values are not sampling weighted

### Measures

*Incident cancer diagnosis.* Incident cancer status was assessed at each biennial study interview in each of the HRS and ELSA as a new self-reported physician diagnosis of cancer, excluding non-melanoma skin cancer. The month and year of the cancer diagnosis were self-reported and used to classify the timing of biennial memory assessments as pre-diagnosis, concurrent with the interview in which the cancer was first reported, and post-diagnosis. Missing dates of cancer diagnosis (18 in ELSA; 43 in HRS) were assigned the mid-point between the last cancer-free interview and the interview when the cancer was reported. Cancer type was self-reported in both the HRS and ELSA to the question, “In which organ or part of your body did this cancer occur?”. Cancer treatment was categorized as any self-reported treatment (yes; no) as ELSA participants are asked if they received any cancer treatment but not what type of cancer treatment.

*Memory function*. Memory function was assessed at each biennial study interview as sum of immediate and delayed recall of a 10-word list, whereby one point was attributed to each correctly recalled word, for a total of 20 points. Respondents represented by a proxy were excluded from the analysis (N = 336; 1.6%). We standardized memory scores at each follow-up time point using the pooled baseline sample mean and standard deviation (SD), therefore analyses present rates of memory change relative to the baseline distribution.

*Covariates.* Potential confounders of the relationship between incident cancer and rate of memory change were assessed at the baseline interview in each cohort (2002) and included: age (years), mean memory function, age- and comorbidity-adjusted mean memory function (derived from model-based adjustment using baseline age and comorbidities), sex/gender (male; female), race (white; non-white); marital status (married/partnered; divorced/separated; widowed; never married), educational attainment (less than high school; high school graduate; some college; college or above), ever smoking, and self-reported comorbidities (hypertension, diabetes, heart disease, stroke (yes; no for each)).

*Statistical analysis.* Differences in baseline characteristics for participants with and without a new cancer diagnosis over the follow-up were described, by study cohort, using chi square statistics for categorical variables and *t* tests for continuous variables. We used sample-weighted multivariable-adjusted linear mixed effects models to model longitudinal memory score trajectories. Following the analytic model of Wang et al., age at each study interview, centered at 75 years, was used as the primary timescale for both those with and without cancer [16]. Individual participants’ slopes of memory change with age and intercepts of memory slope at 75 years of age were allowed to vary as random effects. The covariance structure of random effects was freely estimated using an unstructured covariance specification.

To estimate differences in memory function over time between participants who developed cancer and participants who did not develop cancer. We compared memory trajectories before diagnosis, around the time of diagnosis, and after diagnosis. A time-dependent cancer indicator, which was set to 0 for all time points prior to cancer diagnosis and 1 for all time points at and after diagnosis, accounted for the possible change in memory function around the time of diagnosis. We included an interaction term between continuous time (with respect to cancer diagnosis) and the time-dependent *cancer* variable, allowing for separate slopes in memory in the time prior to a cancer diagnosis and after a cancer diagnosis.

Models included age at cancer diagnosis centered at 75 years (set to 0 for all observations for participants who remained cancer free), which allowed comparison of memory function at the time of diagnosis with that in cancer-free individuals of the same age. Because age at each interview and age of cancer diagnosis were centered at 75 years, the intercept for this model compared the hypothesized memory score in a 75-year-old individual who never had cancer with memory in a 75-year-old individual immediately before he or she was diagnosed with cancer. To test for effect modification by country, we specified a single model using the full sample with interaction terms between each country and each of the cancer-related variables. We then specified models stratified by country and visualized the predicted memory aging slope for cancer-free participants and cancer survivors, by study cohort. Analyses were conducted using StataSE 17.0 (College Station, TX), with data visualizations created in R (Vienna Austria).

We conducted two sensitivity analyses to examine 1) the potential influence of selective attrition following a cancer diagnosis on our results and 2) the potential influence of any cancer treatment on memory function among cancer survivors. We used logistic regression to model study attrition adjusted for baseline age, sex/gender, and study cohort. We then added prediagnosis memory scores to the model to determine whether postdiagnosis attrition was differential by prediagnosis level of memory performance, which would indicate selective survival bias, and if this relationship varied by study cohort, which could indicate varying degrees of attrition bias between the cohorts. We estimated two models to evaluate these relationships, examining two forms of prediagnosis memory scores: (1) baseline memory in 2002, and (2) within-person smoothed memory trajectories before cancer diagnosis, as best unbiased linear predictions from fitted models incorporating all prediagnosis memory observations. To test the association of receiving any cancer treatment on memory function, we restricted the population to only those with an incident cancer diagnosis. We specified linear mixed models stratified by study cohort and whether they self-reported cancer treatment.

## Results

### Sample characteristics

There were 21,616 individuals in our analysis (13,037) in the HRS and 8,579 in ELSA) with 2,777 (12.8%) having an incident cancer diagnosis over the follow-up (1,882 [14.4%] in the HRS and 895 [10.4%] in ELSA; Table 1). Participants were mostly female (57.9%), white (88.4%), and were married or had a partner at baseline (68.6%) (Table 1). Educational attainment significantly differed between participants in the US and England, with more participants in England having less than high school education compared to participants in the US (52.6% vs. 23.6%; Table 1). Compared to participants in England, US participants were slightly older (67.3 years vs. 64.0 years; Table 1) and had more comorbid conditions at baseline including hypertension (48.7% in the US vs. 28.0% in England) and diabetes (15.5% in the US vs. 6.39% in England) (Table 1). Cancer survivors in the US had a higher baseline prevalence of comorbidities than cancer survivors in England (hypertension: 51.9% vs. 28.3%; diabetes: 13.9% vs. 5.82%; Table 2). The overall median follow-up time from baseline in 2002 was 12.2 years (IQR: 6.08—16.0) and was similar in both the HRS (12.2 years; IQR: 6.17–15.9) and ELSA (12.1 years; IQR: 5.75–16.2). After the first incident cancer diagnosis, cancer survivors in the HRS had a slightly longer median follow up time (4.53 years; IQR: 1.62–8.45) than cancer survivors in ELSA (3.96 years; IQR: 1.28–8.29). The distribution of cancer types by country is listed in Supplemental Table 2.

**Table 2:** Baseline Characteristics of the sample, by country and cancer status, in the 2002 Wave of the US Health and Retirement Study (n = 13,037) & the English Longitudinal Study on Ageing (n = 8579)

	Total (N = 21,616)		ELSA (N = 8579)		HRS (N = 13,037)	
	Cancer-free N = 18,839	Incident Cancer N = 2777	Cancer-free N = 7684	Incident Cancer N = 895	Cancer-free N = 11,155	Incident Cancer N = 1882
Age, *years, mean (SD)*	66.1 (9.68)	65.2 (8.10)	64.1 (9.85)	63.5 (8.49)	67.5 (9.32)	66.0 (7.76)
Memory, *mean (SD)*	9.86 (3.56)	10.4 (3.36)	9.60 (3.46)	10.3 (3.32)	10.0 (3.62)	10.5 (3.39)
Age- and comorbidity- adjusted memory,* mean (95% CI)*	9.64 (9.60, 9.67)	10.0 (9.91, 10.1)	10.3 (10.1, 10.4)	9.60 (9.44, 9.75)	10.1 (10.1, 10.2)	9.01 (8.95, 9.08)
Sex/Gender, *n (%)*						
Male	7727 (41.0)	1383 (49.8)	3442 (44.8)	454 (50.8)	4285 (38.4)	929 (49.4)
Female	11,112 (59.0)	1394 (50.2)	4242 (55.2)	441 (49.2)	6870 (61.6)	953 (50.6)
Race, *n (%)*						
White	16,609 (88.2)	2508 (90.3)	7497 (97.6)	884 (98.8)	9095 (81.7)	1624 (86.3)
Non-White	2230 (11.8)	269 (9.6)	187 (2.43)	11 (1.2)	2039 (18.3)	258 (13.7)
Educational Attainment, *n (%)*						
Less than high school	6766 (35.9)	814 (29.3)	4074 (53.0)	436 (48.7)	2692 (25.2)	378 (19.9)
High school	5524 (29.3)	804 (30.0)	1325 (17.2)	151 (16.9)	4191 (37.6)	653 (34.9)
Some college	3630 (19.3)	573 (20.6)	1393 (18.1)	163 (18.2)	2232 (20.1)	410 (21.9)
College and above	2919 (15.5)	586 (21.1)	892 (11.6)	145 (16.2)	2019 (18.1)	441 (23.3)
Marital Status, *n (%)*						
Married/Partnered	12,814 (68.0)	2016 (72.6)	5384 (70.1)	665 (74.4)	7414 (66.6)	1351 (71.8)
Divorced/Separated	1862 (9.87)	281 (10.1)	708 (9.21)	73 (8.17)	1149 (10.3)	208 (11.1)
Widowed	3508 (18.6)	399 (14.4)	1228 (16.0)	117 (13.0)	2280 (20.5)	282 (15.0)
Never Married	655 (3.48)	81 (2.91)	364 (4.74)	40 (4.47)	291 (2.61)	41 (2.18)
Comorbidities, *yes, n (%)*						
Hypertension	7599 (40.4)	1160 (41.8)	2151 (28.0)	254 (28.3)	5442 (48.9)	906 (51.9)
Diabetes	2260 (12.0)	314 (11.3)	496 (6.45)	52 (5.82)	1762 (15.8)	262 (13.9)
Stroke	963 (5.12)	140 (5.04)	263 (3.42)	23 (2.57)	701 (6.30)	117 (6.22)
Heart disease	3364 (17.8)	468 (16.8)	1152 (15.0)	132 (14.8)	2210 (19.9)	336 (17.9)

The cancer-free and incident cancer groups refer to cancer status by the end of the follow-up period in 2018

SD, standard deviation. Values are not sampling weighted

### Linear mixed regression models

**Fig. 2:**
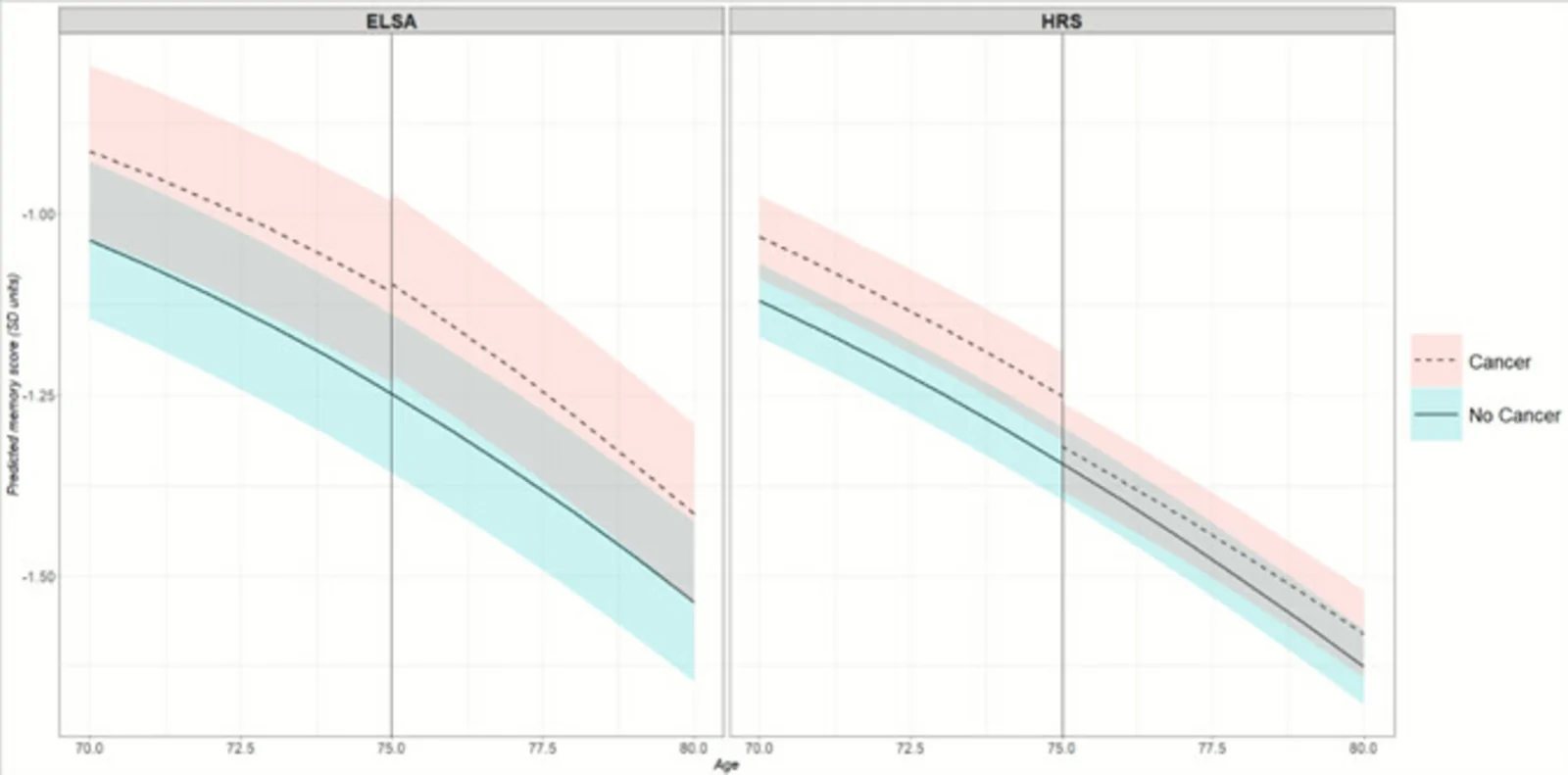
Memory Score Trajectories from Linear Mixed-Effects Models by Country. Predicted memory trajectories from linear mixed-effects models. A. Predicted memory trajectories and 95% CI (shaded area) for an English person diagnosed with cancer at age 75 (vertical line) in the reference categories (male, white, married/partnered, non-smoker, no baseline comorbidities), compared to memory trajectories in a person with the same characteristics but with no cancer diagnosis during the follow-up. B. Predicted memory trajectories in an American person diagnosed with cancer at age 75 in the reference categories, compared to memory trajectories in an American person who remained cancer-free over the follow-up

For cancer-free individuals, the mean memory function at age 75 years was −1.39 SD units (−1.50, −1.28) for ELSA participants and −1.35 SD units (95% CI: −1.41, −1.30) for HRS participants (Table 3; Fig. 2). Cancer-free individuals in both ELSA and HRS had similar mean rates of memory decline over time (−0.51 SD units/decade for an average participant aged 75 years with reference values on all covariates [95% CI: −0.53, −0.49] and −0.53 SD units/decade [95% CI: −0.54, −0.52], respectively; Table 3). Immediately prior to their cancer diagnosis, cancer survivors in both ELSA and the HRS on average had slightly better memory function than cancer-free individuals (0.14 SD units [95% CI: 0.07, 0.20] for ELSA cancer survivors and 0.10 SD units [95% CI: 0.05, 0.14] for HRS cancer survivors; Table 3). At the time of diagnosis, cancer survivors in the HRS experienced a statistically significant drop in memory function that was not seen among cancer survivors in ELSA (−0.08 SD units [95% CI: −0.11, −0.04] for HRS and 0.01 SD units [95% CI: −0.04, 0.07] for ELSA; Table 3). Long-term memory aging slopes in the years before a cancer diagnosis were similar between those with and without an incident cancer diagnosis in both the HRS and ELSA (Table 3; Fig. 2). HRS cancer survivors had a slower rate of memory decline in the years after a cancer diagnosis compared to cancer-free individuals in the HRS (0.06 SD units/decade [95% CI: 0.01, 0.12]; Table 3; Fig. 2) but this was not found among ELSA cancer survivors.

**Table 3:** Estimated association of incident cancer and country on memory function and rate of change using linear mixed models, the US Health and Retirement Study (n = 13,037) & the English Longitudinal Study on Ageing (n = 5879), 2002—2018

	ELSA			HRS		
	β^a^	95% CI	P	β^a^	95% CI	P
**Memory function (SD units) and memory change (SD units/decade)**						
**Participants with no cancer during follow-up**						
Memory function at age 75	−1.39	−1.50, −1.28	0.001	−1.35	−1.41, −1.30	< 0.001
Memory slope with linear age, centered at 75 years	−0.51	−0.53, −0.49	< 0.001	−0.53	−0.54, −0.52	< 0.001
Memory slope with quadratic age (SD units/decade^2^	−0.15	−0.16, −0.15	< 0.001	−0.12	−0.13, −0.11	< 0.001
**Participants with an incident cancer diagnosis during follow-up**						
Difference in memory score right before diagnosis^b^	0.14	0.07, 0.20	0.001	0.10	0.05, 0.14	< 0.001
Acute change in memory at time of diagnosis	0.01	−0.04, 0.07	0.680	−0.08	−0.11, −0.04	< 0.001
Difference in long-term memory slope before diagnosis^b^	0.04	−0.02, 0.11	0.176	0.02	−0.03, 0.07	0.545
Difference in long-term memory slope after diagnosis^b^	−0.04	−0.13, 0.04	0.315	0.06	0.01, 0.12	0.032

^a^Models adjusted for age, age^2^ sex, race, educational attainment, marital status, current smoking, and history of hypertension, diabetes, and stroke, accounted for survey sampling weights

^b^Compared to participants with no cancer over the follow-up, as the reference group

Model specification (person *i* at time *j*): Memory_*ij*_ = β0 + β1 cancer_diagnosis + β2 cancer_now_*j*_ + β3 time_to_diagnosis (zero for time after cancer) + β4 time_since_diagnosis (zero for time before cancer) + β5 age_*j*_ + β6 age^2^_*j*_ + β7 age_at_cancer_diagnosis + ∑βk covariates. Cancer_now_*j*_ is a time-varying binary indicator that equals 0 at time points before diagnosis, and 1 at the time of and after diagnosis

### Sensitivity analyses

A total of 522/1987 of HRS (30.4%) and 280/921 of ELSA participants (26.3%) with cancer stopped contributing data following their reported cancer diagnosis. Higher baseline precancer memory scores were associated with lower likelihood of postdiagnosis attrition (baseline memory: OR = 0.96 per SD unit; 95% CI: 0.93, 0.99 in the pooled sample) but pre-cancer diagnosis memory trajectories were not (OR = 1.37 per SD unit/decade of age; 95% CI: 0.83, 2.24 in the pooled sample). The relationships between prediagnosis memory and postdiagnosis attrition did not vary by county (*P* for interaction = 0.14 for prediagnosis memory score, and 0.35 for prediagnosis memory trajectory), consistent with the notion that selective survival bias conditioned on country and prediagnosis memory does not impact our findings.

Finally, we examined the possible influence of cancer treatment on memory function trajectories in the US and England. Notably, 34.3% of cancer survivors did not self-report any treatment (45.3% in the HRS and 10.5% in ELSA; Supplemental Table 3). The distribution of cancer treatment types for HRS cancer survivors are listed in Supplemental Table 4. Similar to our main analyses, we did not find an acute change in memory at the time of diagnosis for cancer survivors in England regardless of self-reported cancer treatment (Supplemental Table 4). Cancer survivors in the US who reported receiving any type of cancer treatment experienced a drop in memory function at the time of diagnosis (−0.09 SD units; 95% CI: −0.14, −0.04) that was not seen among US cancer survivors reporting no treatment (−0.05 SD units; 95% CI: −0.11, 0.002; Supplemental Table 4).

## Discussion

In this large, population-based and cross-nationally harmonized study of middle-aged and older adults in the United States and England, we found that cancer survivors in the US experienced an acute drop in their memory function around the time of diagnosis that was not experienced by cancer survivors in England. Like others, we observed that cancer survivors had a memory advantage immediately prior to their diagnosis compared to similarly aged cancer-free participants, and this memory advantage was slightly stronger in England than in the US [8]. Long-term prediagnosis and postdiagnosis memory aging trajectories were similar for cancer survivors in both countries. The reasons for our finding of an acute memory drop in the US but not England are not known but could be related to differences in healthcare systems which could differentially impact cancer diagnoses and treatment in both countries.

### Comparison to existing literature

In terms of overall country differences, participants in the ELSA had lower memory levels than HRS participants, regardless of cancer status. Previous research comparing cognitive function across the US and England has been mixed with some studies finding better cognitive function among participants in the US [13], while others finding better cognitive function and lower levels of dementia in England [13, 17, 18]. Fewer studies have compared cognitive function of cancer survivors across the US and England. We found that cancer survivors had better memory function prior to diagnosis compared to cancer-free participants in both countries, also consistent with prior research finding an inverse association between cancer and cognitive aging outcomes including dementia [9]. This paradoxical inverse association has been posited to be due to a potential common cause of cancer and dementia, such as inverse biological regulation of carcinogenesis and neurodegeneration [9]. Other potential explanations include methodological study biases such as selective survival bias, diagnostic bias, competing risk bias, and confounding bias [9]. We found that incident cancer cases who were cognitively healthier in the years before their diagnosis were less likely to die or drop out of the study following diagnosis, but this selective survival pattern was not differential by country and thus could not explain our results.

Also consistent with previous research [8], in the present study cancer survivors in the HRS had a decline in memory function around the time of their diagnosis. This memory decline at diagnosis is thought to be due to the neurotoxic effects of cancer treatment [19–23]. However, we did not see this decline in memory function among cancer survivors in ELSA, a novel finding of this analysis. While the greater prevalence of prediagnosis comorbidities could explain this difference, differences in healthcare systems and social safety nets as well as treatment differences between the two countries could play a role. In England, the National Health Service (NHS) provides publicly funded universal healthcare with a comprehensive range of health services. In contrast, the US lacks universal healthcare with approximately 9% of US adults aged 50 to 64 being uninsured [24]. Most healthcare is provided through private organizations with the exception of programs for low-income (Medicaid) and adults aged ≥ 65 years (Medicare), although healthcare challenges remain for adults aged ≥ 65 in the US [25]. England’s comprehensive universal healthcare and social safety net programs may allow cancer survivors in England to receive earlier cancer diagnoses than those in the US which could preserve better memory function at the time of diagnosis and treatment. More research is needed to better understand the role of the differing healthcare systems on the cancer care continuum and how these differences could influence cognitive health among cancer survivors particularly by socioeconomic status.

Another potential reason for this difference could be due to cancer treatment differences in the US and England, as different treatment regimens could have differential effects on cognitive functioning [5, 26]. A recent study by McPhail and colleagues found that cancer survivors in the United Kingdom were less often treated with chemotherapy and radiotherapy compared to cancer survivors in Canada and Australia [27]. Previous research has demonstrated acute, short-term cognitive declines after cancer treatment including chemotherapy (a phenomenon known as “chemobrain”) [4, 7, 20, 21, 23]. It is possible that the lack of an acute drop in memory function around the time of diagnosis in England is related to the receipt of less aggressive cancer treatments. Indeed, when the analyses were stratified by self-reported treatment, only US cancer survivors who reported any type of cancer treatment had this acute drop in memory function at the time of diagnosis. An important next step is to better understand these potential treatment differences and how type of cancer treatment and timing of treatment could influence memory aging trajectories of older adults in both countries.

### Limitations

This study has limitations. First, cancer diagnoses were self-reported and subject of measurement error. However, previous research has found that the self-reported cancer diagnoses in the HRS have high sensitivity and specificity compared to diagnoses obtained from Medicare claims data [28]. Covariates were self-reported in the HRS and ELSA interviews, and errors in covariate measurement due to these self-reports or interview question limitations could result in residual confounding of the model estimates. There could be bias introduced due to study attrition, as many cancer survivors died soon after diagnosis and our sensitivity analysis showed that attrition was related to prediagnosis memory. However, this relationship was not differential by country and should not affect the cross-national differences we observed. We excluded participants with proxy interviews as they did not have direct memory assessments, which may have resulted in the individuals with the worst memory being excluded. Finally, we did not have cancer clinical characteristics such as stage at diagnosis or treatment information for both cohorts. These factors would be potential mediators of the effect of a cancer diagnosis on memory decline, and should be investigated in future explanatory analyses.

A major strength of this study is its large, nationally representative sample of middle-aged and older adults in both the US and England that used harmonized measures. We had longitudinal data on memory prior to cancer diagnosis, which is often unavailable in clinic-based studies of cancer survivorship that recruit after diagnosis, allowing us to identify the within-person change in memory function associated with a cancer diagnosis. Our sensitivity analyses indicated that post-diagnosis selective survival or study attrition did not explain the cross-country differences in cancer-related memory decline observed in this study.

## Conclusion

Understanding cognitive aging trajectories in the growing population of older cancer survivors will be increasingly important to promote healthy aging in this population. Cross-national comparisons introduce heterogeneity in population-level risk and resilience factors which can allow us to identify drivers of disease at a population level. We observed an acute short term memory decline associated with a cancer diagnosis among cancer survivors in the US, but not England. Future studies should investigate how differences in the healthcare systems in these two countries play a role in cancer diagnoses, treatment delays, and treatment patterns, and how socioeconomic status could modify these associations to further elucidate reasons for these observed differences in memory function.

## Supplementary Information

Below is the link to the electronic supplementary material.

Supplementary file1 (DOCX 34 kb)

## Data Availability

The Health and Retirement Study and English Longitudinal Study on Ageing are publicly available at the following links: https://hrs.isr.umich.edu/ and https://www.elsa-project.ac.uk/
