# Supplementary material for: Cross national comparison of memory function before and after a cancer diagnosis in the United States and England
Source: J Cancer Surviv. 2026 Jan 17;20(Suppl 2):1404–12. doi: 10.1007/s11764-026-01969-8 (PMC12990089; doi:10.1007/s11764-026-01969-8)
Supplement: Supplementary file 1 [file 11764_2026_1969_MOESM1_ESM.docx]

| **Supplemental Table 1. Characteristics of Included and Excluded Participants, Overall and by Country** | | | | | | |
| --- | --- | --- | --- | --- | --- | --- |
|  | **Total** | | **ELSA** | | **HRS** | |
|  | **Included**  **N = 21616** | **Excluded**  **N = 4728** | **Included**  **N = 8579** | **Excluded**  **N = 2712** | **Included**  **N = 13037** | **Excluded**  **N = 2070** |
| Cancer survivors, *n (%)* | 2777 (12.9) | 1630 (34.1) | 908 (10.6) | 808 (29.8) | 1882 (14.4) | 826 (39.9) |
| Age*, mean (SD)* | 66.0 (9.50) | 70.4 (11.1) | 64.0 (9.72) | 67.8 (9.94) | 67.3 (9.12) | 71.3 (8.96) |
| Sex/Gender, *n (%)* |  |  |  |  |  |  |
| Male | 9110 (42.1) | 2263 (47.3) | 3896 (45.4) | 1230 (45.3) | 5214 (39.9) | 1035 (50.0) |
| Female | 12506 (57.9) | 2519 (52.7) | 4683 (54.6) | 1486 (54.7) | 7823 (60.1) | 1035 (50.0) |
| Race, *n (%)* |  |  |  |  |  |  |
| White | 19117 (88.4) | 4291 (89.7) | 8381 (97.7) | 2608 (96.0) | 10736 (82.4) | 1687 (81.5) |
| Non-White | 2499 (11.6) | 487 (10.2) | 198 (2.32) | 108 (3.98) | 2301 (17.7) | 379 (18.3) |
| Unknown | - | 4 (0.08) | - | 0 (0.0) | - | 4 (0.19) |
| Educational Attainment, *n (%)* |  |  |  |  |  |  |
| Less than high school | 7580 (35.1) | 2403 (50.3) | 4510 (52.6) | 1716 (63.2) | 3070 (23.6) | 690 (33.3) |
| High school | 6328 (29.3) | 1020 (21.3) | 1476 (17.2) | 340 (12.5) | 4852 (37.2) | 680 (32.9) |
| Some college | 4203 (19.4) | 725 (15.2) | 1556 (18.1) | 369 (13.6) | 2647 (20.3) | 357 (17.3) |
| College and above | 3505 (16.2) | 571 (11.9) | 1037 (12.1) | 233 (8.58) | 2468 (18.9) | 338 (16.3) |
| Unknown | - | 63 (1.32) | - | 58 (2.14) | - | 5 (0.24) |
| Marital Status, *n (%)* |  |  |  |  |  |  |
| Married/Partnered | 14830 (68.6) | 2970 (62.1) | 6049 (70.5) | 1783 (65.7) | 8781 (67.4) | 1190 (57.5) |
| Divorced/Separated | 2143 (9.91) | 392 (8.20) | 781 (9.10) | 209 (7.7) | 1362 (10.5) | 183 (8.84) |
| Widowed | 3907 (18.1) | 1182 (24.7) | 1345 (15.7) | 558 (20.5) | 2562 (19.7) | 625 (30.2) |
| Never Married | 736 (3.40) | 221 (4.62) | 404 (4.7) | 164 (6.04) | 332 (2.55) | 57 (2.75) |
| Unknown | - | 17 (0.36) | - | 2 (0.07) | - | 15 (0.72) |
| Hypertension, *n (%)* |  |  |  |  |  |  |
| Yes | 8759 (40.5) | 2050 (42.9) | 2405 (28.0) | 882 (32.5) | 6354 (48.7) | 1171 (56.6) |
| Missing | - | 9 (0.19) | - | 8 (0.29) | - | 1 (0.05) |
| Diabetes, *n (%)* |  |  |  |  |  |  |
| Yes | 2574 (11.9) | 711 (14.9) | 548 (6.39) | 256 (9.43) | 2026 (15.5) | 455 (21.9) |
| Missing | - | 5 (0.10) | - | 4 (0.15) | - | 1 (0.05) |
| Stroke, *n (%)* |  |  |  |  |  |  |
| Yes | 1103 (5.10) | 444 (9.28) | 286 (3.33) | 162 (5.96) | 817 (6.27) | 283 (13.7) |
| Missing | - | 5 (0.1) | - | 4 (0.15) | - | 1 (0.05) |
| Heart disease, *n (%)* |  |  |  |  |  |  |
| Yes | 3832 (17.7) | 1322 (27.7) | 1284 (15.0) | 621 (22.9) | 2548 (19.5) | 702 (33.9) |
| Missing | - | 5 (0.11) | - | 4 (0.15) | - | 1 (0.05) |
| Self-report any cancer treatment, *yes, n (%)* | 1807 (65.7) | 990 (63.0) | 779 (89.5) | 412 (55.3) | 1028 (54.6) | 578 (70.0) |
| Missing | 25 (0.90) | 59 (3.62) |  | 59 (7.34) |  |  |
| Cancer type, *n (%)* |  |  |  |  |  |  |
| Bronchia; bronchus; lung; ‘chest’ | 245 (8.82) | 75 (4.53) | 57 (5.6) | 18 (2.24) | 188 (10.0) | 57 (6.69) |
| Breast | 464 (16.7) | 360 (21.7) | 150 (16.8) | 235 (29.2) | 314 (16.7) | 125 (14.7) |
| Bowel; Colon; Rectum | 308 (11.1) | 176 (10.6) | 125 (14) | 101 (12.6) | 183 (9.7) | 75 (8.8) |
| Lymphoma | 58 (2.09) | 32 (1.93) | 24 (2.7) | 22 (2.74) | 34 (1.8) | 10 (1.17) |
| Leukemia | 75 (2.70) | 22 (1.33) | 6 (0.7) | 8 (1.0) | 69 (3.7) | 14 (1.64) |
| Melanoma | 158 (5.69) | 71 (4.29) | 147 (16.4) | 65 (8.08) | 11 (0.6) | 6 (0.70) |
| Prostate | 512 (18.4) | 184 (11.1) | 129 (14.4) | 6 (0.75) | 383 (20.4) | 178 (20.9) |
| Other site | 875 (31.5) | 520 (31.4) | 232 (25.9) | 288 (35.8) | 643 (34.2) | 232 (27.2) |
| Unknown/Missing | 82 (2.95) | 216 (13.3) | - | 61 (7.59) | 57 (3.03) | 155 (18.8) |

**Supplemental Table 2. Self-reported cancer site by country**

|  | **Self-Reported Cancer Site** | |
| --- | --- | --- |
|  | **ELSA (n=895)** | **HRS (n=1882)** |
| Bronchia; bronchus; lung; ‘chest’ – NSF | 57 (6.4) | 188 (10.0) |
| Breast | 150 (16.8) | 314 (16.7) |
| Bowel; Colon; Rectum | 125 (14) | 183 (9.7) |
| Lymphoma | 24 (2.7) | 34 (1.8) |
| Leukemia | 6 (0.7) | 69 (3.7) |
| Melanoma | 147 (16.4) | 11 (0.6) |
| Prostate | 129 (14.4) | 383 (20.4) |
| Other site | 232 (25.9) | 643 (34.2) |
| Missing | 25 (2.8) | 39 (2.1) |
| Unknown (refused) | 0 (0) | 18 (1.0) |

**Supplemental Table 3. Baseline Characteristics of cancer survivors by self-reported cancer treatment, overall and by country, in the 2002 wave of the US Health and Retirement Study (HRS; n=1882) & the English Longitudinal Study on Ageing (ELSA; n=870)**

|  | **Total** | | **ELSA (n = 870)** | | **HRS (n=1882)** | |
| --- | --- | --- | --- | --- | --- | --- |
|  | **Self-Reported Treatment** | | **Self-Reported Treatment** | | **Self-Reported Treatment** | |
|  | **No**  **N=945** | **Yes**  **N=1807** | **No**  **(n=91; 10.5%)** | **Yes**  **(n=779; 89.5%)** | **No**  **(n=854; 45.4%)** | **Yes**  **(n=1028; 54.6%)** |
| Age, *years, mean (SD)* | 66.8 (8.38) | 64.6 (7.95) | 63.6 (8.55) | 63.4 (8.28) | 67.0 (8.23) | 65.4 (7.48) |
| Memory, *mean (SD)* | 10.2 (3.38) | 10.5 (3.35) | 10.5 (3.34) | 10.1 (3.22) | 10.3 (3.36) | 10.5 (3.38) |
| Sex/Gender, *n (%)* |  |  |  |  |  |  |
| Male | 461 (48.8) | 911 (50.4) | 51 (56.0) | 392 (50.3) | 410 (48.0) | 519 (50.5) |
| Female | 484 (51.2) | 896 (49.6) | 40 (43.9) | 387 (49.7) | 444 (52.0) | 509 (49.5) |
| Race, *n (%)* |  |  |  |  |  |  |
| White | 821 (86.9) | 1662 (91.9) | 90 (98.9) | 769 (98.7) | 731 (85.6) | 893 (86.9) |
| Non-White | 124 (13.1) | 145 (8.02) | 1 (1.10) | 10 (1.28) | 123 (14.4) | 135 (13.1) |
| Educational Attainment, *n (%)* |  |  |  |  |  |  |
| Less than high school | 238 (25.2) | 563 (31.0) | 57 (62.6) | 366 (46.9) | 181 (21.2) | 197 (19.2) |
| High school | 478 (27.5) | 478 (26.5) | 11 (12.1) | 134 (17.2) | 309 (36.2) | 344 (33.5) |
| Some college | 377 (20.9) | 377 (20.9) | 14 (15.4) | 146 (18.7) | 179 (21.0) | 231 (22.5) |
| College and above | 389 (21.5) | 389 (21.5) | 9 (9.89) | 133 (17.1) | 185 (21.7) | 256 (24.9) |
| Marital Status, *n (%)* |  |  |  |  |  |  |
| Married/Partnered | 661 (70.0) | 1335 (73.9) | 69 (75.8) | 576 (73.9) | 592 (69.3) | 759 (73.8) |
| Divorced/Separated | 102 (10.8) | 177 (9.8) | 6 (6.59) | 65 (8.34) | 96 (11.2) | 112 (10.9) |
| Widowed | 152 (16.1) | 245 (13.6) | 13 (14.3) | 102 (13.1) | 139 (16.3) | 143 (13.9) |
| Never Married | 30 (3.17) | 50 (2.77) | 3 (3.3) | 36 (4.62) | 27 (3.16) | 14 (1.36) |
| Comorbidities, *yes, n (%)* |  |  |  |  |  |  |
| Hypertension | 444 (47.0) | 706 (39.1) | 23 (25.3) | 221 (28.4) | 421 (49.3) | 485 (47.2) |
| Diabetes | 137 (14.5) | 176 (9.74) | 8 (8.79) | 43 (5.52) | 129 (15.1) | 133 (12.9) |
| Stroke | 59 (6.24) | 79 (4.37) | 3 (3.30) | 18 (2.31) | 56 (6.56) | 61 (5.93) |
| Heart disease | 193 (20.4) | 268 (14.8) | 14 (15.4) | 111 (14.3) | 179 (21.0) | 157 (15.3) |
| Abbreviations: SD, standard deviation. Values are not sampling weighted. | | | | | | |

**Supplemental Table 4. Self-report cancer treatment for HRS participants with an incident cancer diagnosis**

| **Treatment Reported, yes** | **HRS incident cancer (n=1882), N (%)** |
| --- | --- |
| Chemotherapy | 440 (23.4) |
| Surgery | 566 (30.1) |
| Radiation | 367 (19.5) |
| Medication | 218 (11.6) |
| Biopsy | 59 (3.13) |
| X-ray | 26 (1.38) |
| Other treatment | 118 (6.27) |

**Supplemental Table 5. Association of any cancer treatment on memory function and rate of change using linear mixed models, the US Health and Retirement Study (n=1882) & the English Longitudinal Study on Ageing (n=895), 2002 - 2018**

|  | **ELSA** | | **HRS** | |
| --- | --- | --- | --- | --- |
|  | **Self-Reported Treatment** | | **Self-Reported Treatment** | |
|  | **No** | **Yes** | **No** | **Yes** |
| Memory function at age 75 | -0.28 (-1.70, 1.15) | -1.23 (-1.65, -0.81) | -1.05 (-1.24, -0.85) | -1.31 (-1.48, -1.13) |
| *Entire period* |  |  |  |  |
| Memory slope with linear age, centered at 75 years | -0.38 (-0.60, -0.16) | -0.45 (-0.52, -0.38) | -0.45 (-0.51, -0.40) | -0.42 (-0.47, -0.36) |
| *Pre and Post Cancer Periods* |  |  |  |  |
| Acute change in memory at time of diagnosis | -0.02 (-0.20, 0.16) | 0.01 (-0.05, 0.07) | -0.05 (-0.11, 0.002) | -0.09 (-0.14, -0.04) |
| Pre-cancer diagnosis memory slope | -0.14 (-0.39, 0.11) | -0.003 (-0.09, 0.08) | -0.08 (-0.17, 0.01) | -0.04 (-0.12, 0.06) |
| Post-cancer diagnosis memory slope | -0.41 (-0.76, -0.05) | -0.06 (-0.16, 0.04) | -0.04 (-0.13, 0.05) | -0.02 (-0.10, 0.07) |
| Models adjusted for age, age^2^ sex, race, educational attainment, marital status, current smoking, and history of hypertension, diabetes, and stroke, accounted for survey sampling weights . | | | | |
